# Detection of Propionic Acids Trapped in Thin Zeolite Layer Using Thermal Desorption Analysis

**DOI:** 10.3390/s23177352

**Published:** 2023-08-23

**Authors:** Giuseppe Oliva, Antonino S. Fiorillo, Syed Kamrul Islam, Salvatore A. Pullano

**Affiliations:** 1 Biomedical Applications Technologies & Sensors (BATS) Laboratory, Department of Health Sciences, Magna Græcia University of Catanzaro, Viale Europa, 88100 Catanzaro, Italy; giuseppe.oliva@unicz.it; 2 Department of Electrical Engineering and Computer Science, University of Missouri, Columbia, MO 65211, USA; islams@missouri.edu

**Keywords:** nanoporous materials, thin zeolite layer, zeolite sensor, photoionization technique, thermal desorption processes, molecular sieve

## Abstract

Volatile organic compounds (VOCs) have recently received considerable attention for the analysis and monitoring of different biochemical processes in biological systems such as humans, plants, and microorganisms. The advantage of using VOCs to gather information about a specific process is that they can be extracted using different types of samples, even at low concentrations. Therefore, VOC levels represent the fingerprints of specific biochemical processes. The aim of this work was to develop a sensor based on a photoionization detector (PID) and a zeolite layer, used as an alternative analytic separation technique for the analysis of VOCs. The identification of VOCs occurred through the evaluation of the emissive profile during the thermal desorption phase, using a stainless-steel chamber for analysis. Emission profiles were evaluated using a double exponential mathematical model, which fit well if compared with the physical system, describing both the evaporation and diffusion processes. The results showed that the zeolite layer was selective for propionic acid molecules if compared to succinic acid molecules, showing linear behavior even at low concentrations. The process to define the optimal adsorption time between the propionic acid molecules was performed in the range of 5 to 60 min, followed by a thermal desorption process at 100 °C. An investigation of the relationship between the evaporation and diffusion rates showed that the maximum concentration of detected propionic acid molecules occurred in 15 min. Other analyses were performed to study how the concentration of VOCs depended on the desorption temperature and the volume of the analysis chamber. For this purpose, tests were performed using three analysis chambers with volumes of 25 × 10^−6^, 50 × 10^−6^, and 150 × 10^−6^ m^3^ at three different desorption temperatures of 20 °C, 50 °C, and 100 °C, respectively. The results demonstrated that the evaporation rate of the VOCs increased rapidly with an increasing temperature, while the diffusion rate remained almost constant and was characterized by a slow decay time. The diffusion ratio increased when using a chamber with a larger volume. These results highlight the capabilities of this alternative technique for VOC analysis, even for samples with low concentrations. The coupling of a zeolite layer and a PID improves the detection selectivity in portable devices, demonstrating the feasibility of extending its use to a wide range of new applications.

## 1. Introduction

**Figure 1:**
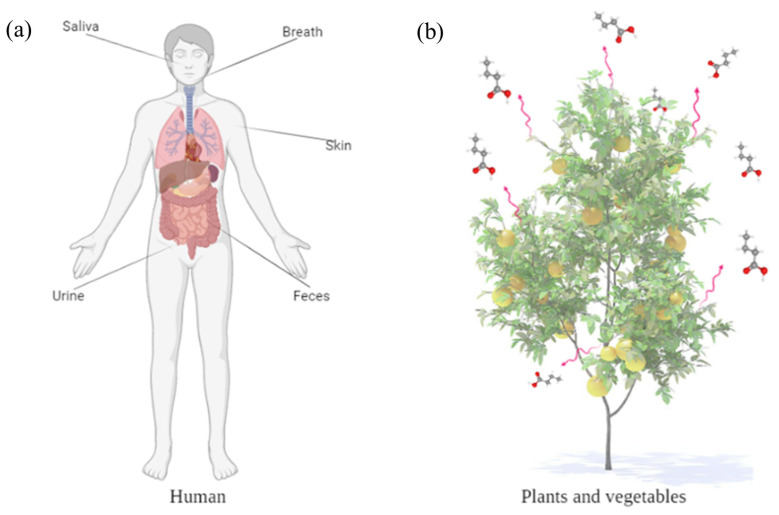
(**a**) Non-invasive sources of VOCs in humans. (**b**) Emission of VOCs by plants and vegetables during functional and/or stimulated processes.

Volatile organic compounds (VOCs) can be classified according to the specific source of production and represent a wide class of chemical compounds characterized by different chemical/physical activities and a peculiar high volatility [1,2,3]. They are directly involved in various biochemical and biological processes that occur in humans, microorganisms, and plants and can be employed as biomarkers [4,5,6,7]. Propionic acid belongs to this class, and its presence in humans has been correlated with a reduction in fatty acid in the liver, resulting in a beneficial effect in obesity and type 2 diabetes [8]. Moreover, other processes such as inflammatory and oxidative stress, etc., produce differently expressed specific VOCs. For human samples, saliva contains about 550 different VOCs that represent 15% of the human volatilome (Figure 1a) [9]. Among them, lactones, ketones, alcohols, halogenated hydrocarbons, and organic acids are the most common chemical species [10]. Organic short-chain fatty acids such as propionic, succinic, and butyric acids, etc., are among the main end products of processes that occur in glands and the microbial fermentation of carbohydrates in the stomach [11,12]. They are also involved in the modulation of cytokines during inflammatory processes [13,14,15]. In addition, a correlation has been evidenced between endogenous VOCs expressed in saliva and those in blood. These can be attributed to the transport mechanisms such as diffusion, ultrafiltration, etc., highlighting the interconnection between different biological samples [16]. Similarly, in plants, VOCs are involved in and strongly influence a variety of processes such as growth and defense mechanisms, abiotic stress, insect/pest resistance, etc. [17,18], as illustrated in Figure 1b.

Standard techniques for VOC detection include the gas chromatography (GS) separation of samples in the gas phase, often associated with mass spectrometry (GS-MS) [19,20]. It requires the pre-processing of the samples, restricting its use in specialized laboratory settings. An alternative consists of a photoionization detector (PID) based on a high-intensity light source (ultraviolet light) with wavelengths lower than 143 nm, which is capable of ionizing molecules and generating a photocurrent proportional to the VOC concentration. A PID can detect both organic and inorganic molecules, but, due to a low detection limit and poor selectivity, they are used in semi-quantitative portable devices for the analysis of complex samples or in association with gas chromatography (GC-PID) [21,22]. Thus, a major limit of PIDs is their capability to be selective with respect to specific molecules, especially if they are chemically similar (e.g., similar ionization energy) as in the case of propionic and succinic acids.

The improvement of the detection selectivity in portable devices such as PIDs can allow a wider range of new applications with human and biological samples, enhancing the monitoring of biological processes through VOC detection. Zeolite is a microporous aluminosilicate with a peculiar crystalline structure, a specific Si/Al ratio, and a mobile metal cation, which confers stable chemical/physical characteristics [23,24,25]. One of the main characteristics of this porous material is its physical adsorption capability, which allows the trapping of specific molecules inside the crystalline framework in the liquid or gaseous phases [26,27,28,29]. Recent studies evidenced that zeolite is found in a wide range of environmental applications, such as reducing VOC levels, the removal of CO_2_ from the environment, etc. [30]. Exploiting the molecular sieve characteristics, we investigated Linde Type A (LTA) zeolite for the fabrication of a thin, porous layer capable of selectively adsorbing propionic acid. In particular, the porous layer made by a mixture of zeolite 4A and soybean oil, deposited onto a silicon substrate, has been used as a molecule-specific thermal desorption device. VOCs are identified and quantified through the evaluation of emission profiles during the thermal desorption phase. An analytical technique combining selective thermal desorption and photoionization detection for the detection of propionic acid is presented. The aim is to provide a zeolite-modified PID to improve its performances, similar to the use of combined techniques.

## 2. Materials and Methods

Synthetic zeolite LTA (4A) is a porous material with a crystallin structure composed of tetrahedral [SiO_4_]^4−^ and [AlO_4_]^5−^ building blocks, with each unit having four equilateral triangles. The spatial arrangement of the two tetrahedral units affects the type of crystallinity of the structure. A tetrahedron possesses four vertices, and the union of one Si tetrahedron and one Al tetrahedron takes place through a shared oxygen atom. In the case of zeolite LTA, tetrahedra are arranged in a structure called a β-cage, which is formed by 6 four-membered rings and 8 six-membered rings. Finally, β-cages and four-membered rings can arrange together to form a basic unit of zeolite, such as LTA, which is included inside a so-called α-cage [31]. The latter contains an aperture composed of eight-membered rings, and in this case the Si/Al ratio is close to 1. This ratio indicates that the framework is negatively charged, and balancing is performed using positive charges (cations), which influence the overall openings of the structure. If the counteranion is potassium, zeolite is identified as 3A with openings of about 3 Å, while a smaller counteranion such as sodium will lead to zeolite 4A, which is characterized by wider openings of about 4 Å. Subsequently, a mobile metal cation such as calcium (Ca^2+^), compensating two anions, will provide much wider openings of about 5 Å. In this study, Zeolite 4A, furnished by UOP Honeywell, Reggio Calabria, Italy, was employed. Zeolite 4A powder is characterized by cubic grains measuring 450 nm with a density of 700 mg/mL, a pH of 11.00, and the presence of mobile Na cations. These structural characteristics of the zeolite make it possible to define a nanoporous framework to be used in different applications such as molecular sieves and ion exchangers [32].

Soybean oil is composed of 55.4% 9,12-octadecadienoic acid, 26.3% 9-octadecenoic acid, 12.8% hexadecanoic acid, 4.2% octadecanoic acid, and 1.3% 12-octadecenoic acid. Its principal chemical and physical characteristics are represented by a smoke point of 133 °C, a low viscosity (3.4 × 10^−5^ m^2^/s), a high iodine value (i.e., 124), and a low flash point (250 °C). Its composition and high iodine value create a carbonic matrix during the annealing process, allowing the formation of a compact layer with embedded zeolite particles.

Organic compounds belonging to short-chain fatty acids are characterized by the carboxyl group (-COOH), a hydroxyl group (-OH) bound to a carbonyl group (-C=O). Carboxylic acids are polar molecules capable of creating hydrogen links, both receiving with the -C=O group and giving with the -OH group. Moreover, they can easily form hydrogen bonds with the same or different molecular types, resulting in an interesting model system for many biological structures such as peptides, amino acids, etc. [33]. Among them, we used propionic acid (C3H6O2) provided by Sigma Aldrich (99% pure liquid solution), characterized by a molar weight of 74.08 g/mol and a framework with a larger size of about 3.6 Å. Moreover, succinic acid (C4H6O4) provided by Sigma Aldrich (99% pure powder solution) was characterized by a molar weight of 118.09 g/mol and a framework with a larger size of about 5.2 Å. 

A mixture of 60% *w*/*w* zeolite 4A and 40% *w*/*w* soybean oil was prepared using an Ultra-Turrax homogenizer at 5000 rpm for 10 min. For the realization of the layer, a silicon substrate with dimensions of 18 × 18 mm, used as support, was covered with a zeolite mixture, and subsequently the mixture was deposited on it using a spin-coating technique at 3000 rpm for 60 s.

The composition allowed us to obtain a mixture with a viscosity that allowed a controllable deposition using a spin-coating technique. The silicon sample was positioned on a hot plate at 200 °C for 3 h so that the soybean oil decomposed, making a carbon matrix capable of keeping the individual zeolite grains together and attached to the silicon substrate. 

**Figure 2:**
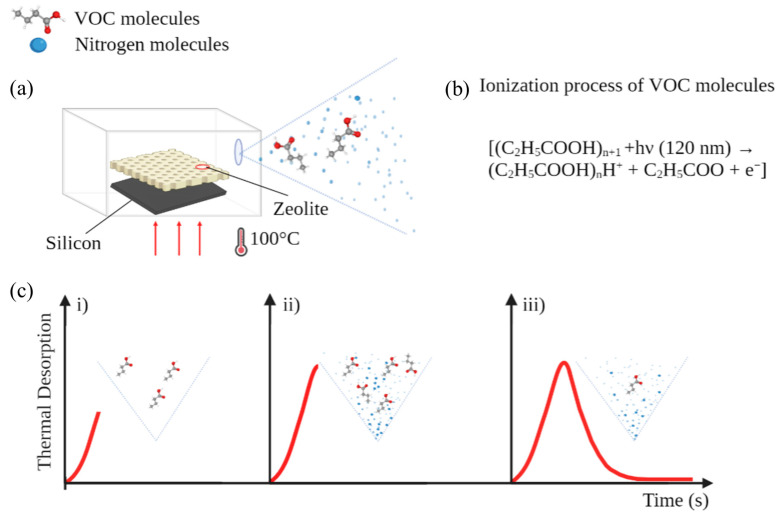
(**a**) Schematic of thermal desorption process of zeolite-based system; (**b**) ionization processes of propionic acid molecules through UV stimulation; (**c**) analysis of emissive profiles during the thermal desorption processes: (**i**) phase of increase in VOC emission, (**ii**) maximum peak of VOC emission, (**iii**) end of VOC emission.

As a result of this process, a profilometric analysis (Veeco, Dektak 6M) evidenced a nanoporous layer with a thickness of 20 μm composed of individual grains of zeolite 4A with a size of 450 nm. In addition, the analysis highlighted an average layer roughness of 1.3 µm. Considering the dimensional characteristics of the nanoporous layer and the density of zeolite 4A, it appeared that in the layer there was about 7 mg of zeolite. Before the adsorption process, the zeolite layer was placed on the hot plate at 100 °C for 5 min to remove water molecules from the pores and improve the adsorption capacity of the nanoporous layer. Then, 2 mL of propionic and succinic acids at different molar concentrations were adsorbed for 5 min. Starting from the solutions of propionic and succinic acids at 99.9% *v*/*v*, samples with variable concentrations in the range of 13.36 M–13.3 mM were obtained. One of the features of VOCs is their state transition in the gas form, which can occur spontaneously at ambient temperature (about 20 °C) and is strongly influenced by increasing temperatures. Adsorption/desorption processes were monitored inside stainless-steel chambers with volumes of 25 × 10^−6^, 50 × 10^−6^, and 150 × 10^−6^ m^3^ to reduce the interactions between the zeolite layer and the external environment during the adsorption process since it is inert and does not release VOCs. Following the adsorption process, the zeolite layer was rinsed with deionized water and dried with a nitrogen (N_2_) flow to remove the residuals of molecules on the layer’s external surface. An analysis of the VOCs was performed at controlled temperatures of 20, 50, and 100 °C inside a chamber with a specific volume. The chamber was equipped with two inlet/outlet holes with diameters of 3 mm for the introduction of external gas and connection to the photoionizer. The design approach used in this work consisted of the development of a customized analytical separation technique using a zeolite-based sensor combined with a photoionization system. The concentration was expressed in parts per million (ppm) of specific VOCs calibrated against isobutylene, which was the gas used for the calibration of the PID. The analysis procedure started when the zeolite layer was inserted inside the chamber of analysis (t = 0) and lasted for 5 min (Figure 2a). 

The VOC detection system was based on the use of a commercial photoionizer (MiniRAE 3000, Recom Industriale s.r.l., Genova, Italy) that included an ultraviolet (UV) lamp with a high energy of 10.6 eV and a wavelength of 120 nm. The system was capable of ionizing VOC molecules present in the desorption chamber, thereby creating an electrical current correlated with the molecules per unit of volume (Figure 2b). In gas detectors, such as a PID, the ionization of molecules appears as electron–ion pairs in which these charge carriers are attracted through the anode and the cathode due to the electric field applied between the electrodes. The generated current signal is correlated with the number of ionized molecules per unit of volume, expressed as a ppm (parts per million) value. Measurements are performed in the range of 0.1 ppm to 15,000 ppm. 

Emission profiles were offline and were analyzed using MATLAB^TM^ (MathWorks, Natick, MA, USA). Analyses of the emissive profiles of VOCs can be mathematically modeled considering the evaporation and diffusion processes, as reported in the literature [34,35]. The concentration of VOCs in a close environment is ruled by two physical processes that occur during the thermal desorption process, specifically partial evaporation (Figure 2c(i,ii)) and partial diffusion (Figure 2c(iii)). The processes are modeled using a nonlinear double exponential function to analyze the emission rate of VOCs as

(1) $$C\left(t\right)=E^{-k1t}+D^{-k2t}$$

where *C*(*t*) represents the total emission ratio of thermally desorbed VOCs (mg/m^2^·min), *E* (mg/m^2^·min) represents the VOC emission rate controlled by the evaporation process, and *D* (mg/m^2^·min) represents the VOC emission rate controlled by the diffusion process. Finally, *k*_1_ (s^−1^) is the evaporation coefficient, which depends on the properties of the liquid and the environmental conditions such as temperature and humidity, while *k*_2_ (s^−1^) is the emission decay constant during the diffusion process, which mainly depends on the size of the system; the viscosity of the medium; the properties of the diffusing substance (zeolite), such as the porosity and density of zeolite; and the velocity of the air present inside the analysis system. 

The mechanism that occurs during the emission phase (E), represents the start of the desorption dynamic in the short period immediately after the adsorption process. The evaporation process takes place rapidly and is characterized from a rapid emission of the VOCs released from the zeolite layer, as shown in Figure 2c(i), that evidences a fast rise time in the emissive profile followed by a plateau phase (Figure 2c(ii)). This is the rapid evacuation of the VOCs from inside the pores of zeolite, which subsequently varies the concentration of the VOCs in the chamber volume. During the diffusion phase (D), the zeolite layer is nearly completely devoid of the VOCs inside it, as evidenced by the representative model shown in Figure 2c(iii), characterized by a profile with a slow fall time. In these processes, the zeolite layer represents the vector that leads the adsorption/desorption process. Considering the balance of concentration detected in the chamber by integrating the parameters defined in Equation (1) and assuming the initial concentration is equal to zero, it can be written as follows:

(2) $$C=L\left[\frac{E\left(e^{-k1t}-e^{-nt}\right)}{n-k1}+\frac{D\left(e^{-k2t}-e^{-nt}\right)}{n-k2}\right]$$

where L is the material load factor that represents the capability of the zeolite sample to trap the VOC, expressed as the ratio between the area and volume of the zeolite layer (m^2^/m^3^), and n is expressed as the ratio between the nitrogen flows (m^3^/s) inside the volume (m^3^) of the chamber of analysis. Data retrieved from the PID, expressed in parts per million in volume (ppmv) were converted to match those reported in Equation (2) expressed in mg/m^3^. To this end, the volumetric concentration (Vc) was expressed as the product between the molar volume ($V_{m}$) and the corrective factor (CF), divided by the molecular weight (M_W_) of the specific molecule being considered. Both evaporation and diffusion processes were described by an exponential model based, respectively, on the reduction in the liquid amount and the diffusion of a substance from one point to another inside the system.

## 3. Results and Discussion

**Figure 3:**
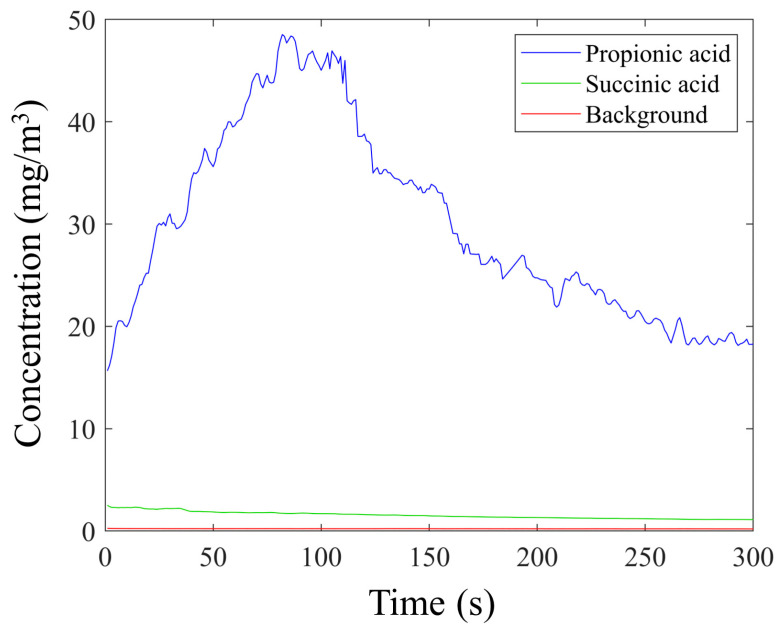
Emissive profiles detected by PID of bare zeolite layer (background) and after the adsorption process with propionic and succinic acids.

Preliminary tests were conducted inside the chamber of analysis with a volume of 150 × 10^−6^ m^3^ after 60 min of zeolite layer immersion in 2 mL of a pure sample solution of propionic and succinic acids. The thermal desorption was performed using a controlled temperature of 100 °C. The model reported in Equation (2) was used to describe the emission of VOCs that occurred inside the chamber during the thermal desorption process. The background emission level of the zeolite layer is shown in Figure 3, and no significant release of molecules was detected. At the same time, the emission profiles of succinic and propionic acids were quite different.

**Figure 4:**
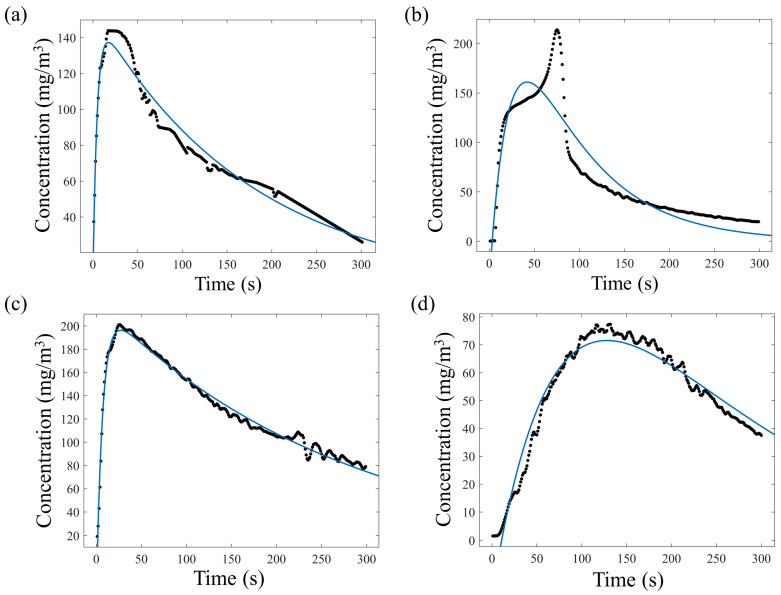
VOC emissive profiles and nonlinear regressions with exposure times of (**a**) 5 min, (**b**) 15 min, (**c**) 30 min, and (**d**) 60 min.

The reason for this different adsorption and thus desorption can be due to the different sizes of the molecules and the pore size of zeolite 4A. Specifically, the size of a succinic acid molecule (5.2 Å) is bigger than the pore size of zeolite (4 Å), and thus they are size-excluded from the nanoporous layer. The residual level of succinic acid detected can be attributed to the residual molecules still bound to the surface. The molecular size of propionic acid (3.6 Å) is compatible with the pore size of the zeolite. In addition, by comparing the emission profiles of Figure 3, the detected concentration peak of propionic acid molecules was about 50 times higher than that of succinic acid. The data evidenced how zeolite 4A can selectively adsorb these two VOCs, and therefore the results reported below will focus on the characterization of the emissive profile of propionic acid considering the mathematical model reported in Equation (2). The first analysis was conducted using a sample solution containing 6.65 M propionic acid, which was brought into contact with the nanoporous layer for 5, 15, 30, and 60 min in a chamber measuring 50 × 10^−6^ m^3^ at a temperature of 100 °C. Figure 4 shows the emission profile and the nonlinear regression using the model in Equation (2). The resulting parameters are also reported in Table 1. Tests were performed using the measurement conditions previously described and considering the same adsorption capability of the zeolite layer (L). In addition, the constant “n” was expressed as the nitrogen flow inside the analysis chamber that resulted in a flow of 0.5 L/min, which is equal to the volume sampled by the photoionizer during the analysis.

**Figure 5:**
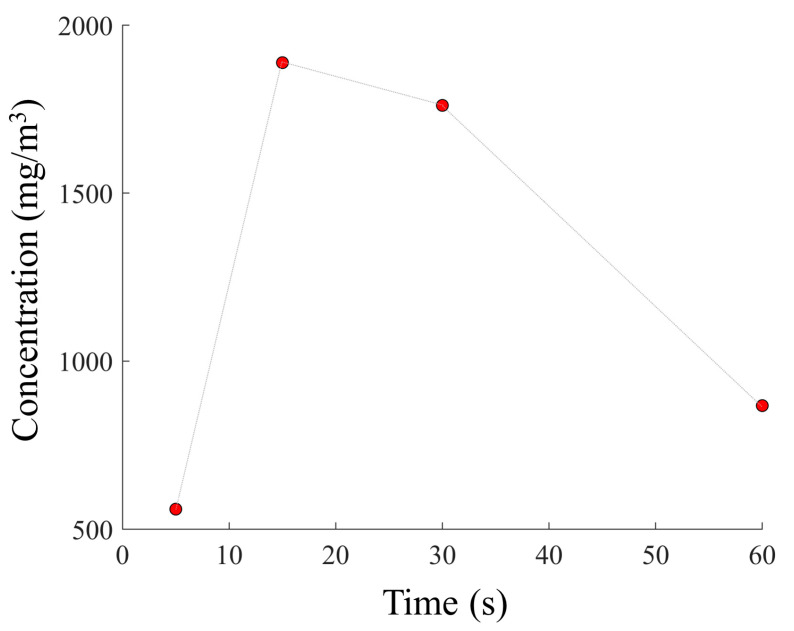
Total concentrations of propionic acid molecules versus adsorption time of a 6.65 M solution of propionic acid. The red markers depict *E/k*_1_ + *E/k*_2_.

The regression showed that the dynamics of the emission profiles were strongly correlated with the evaporation and the diffusion phases, characterized, respectively, by evaporation (*k*_1_) and diffusion (*k*_2_). It was evidenced that the *E/k*_1_ ratio provides information about the total VOCs released during the evaporation phase and the quantities of molecules previously adsorbed inside the layer. Similarly, the *D/k*_2_ ratio represents the total VOCs present inside the volume of the analysis chamber during the diffusion phase. A quantification of the total propionic acid molecules previously adsorbed within the zeolite layer and released into the considered volume can be monitored by considering *E/k*_1_ + *D/k*_2_ (Table 1), as graphically shown in Figure 5. Therefore, the higher the ratios of *E/k*_1_ and *D/k*_2_, the greater the number of molecules present within the layer. The results demonstrate that using an adsorption time of 15 min, the total concentration of VOCs detected was higher if compared with adsorption times of 5, 30, and 60 min (Figure 5).

**Figure 6:**
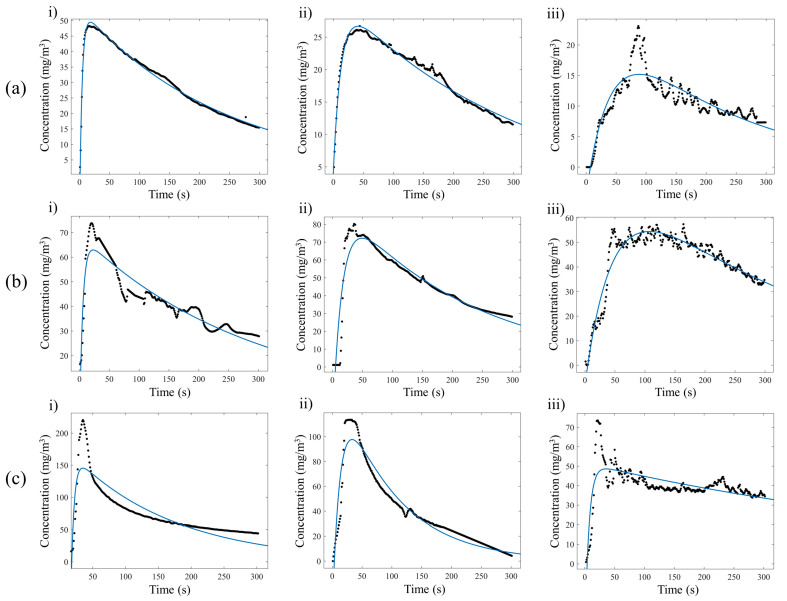
Desorption dynamics of zeolite 4A layer adsorbed with a 6.65 M diluted solution of propionic acid using analysis chamber volumes of (**i**) 25 × 10^−6^ m^3^, (**ii**) 50 × 10^−6^ m^3^, and (**iii**) 150 × 10^−6^ m^3^ and temperatures of (**a**) 20 °C, (**b**) 50 °C, and (**c**) 100 °C.

A significant decrease in the VOC concentration was observed using an adsorption time of 60 min. The reduction in the concentration of the detected VOCs beyond the optimal adsorption time can be attributed to external environmental factors, such as temperature, and the propionic acid molecules in the sample solution. VOCs are highly volatile, and after 30 min of adsorption, a spontaneous desorption process could overcome the number of adsorbed molecules. As previously highlighted, it is important to consider the dimensions of the analysis chamber and the temperature during the thermal desorption process. Three analysis chambers with volumes of 25 × 10^−6^, 50 × 10^−6^, and 150 × 10^−6^ m^3^ were investigated. Each of these was subjected to controlled temperatures of 20 °C, 50 °C, and 100 °C, as reported in Figure 6a–c, highlighting how the desorption dynamics and consequently the interpretation related to the detection of VOCs change. The changes in the emissive dynamics of VOCs were investigated in terms of the evaporation decay constant (1/*k*_1_) and the diffusion decay constant (1/*k*_2_), as reported in Table 2.

To evaluate the impact of these factors, the time constants 1/*k*_1_ (s) and 1/*k*_2_ (s), which represent the growth time relative to the evaporation phase and the decay time relative to the diffusion phase, were considered.

**Figure 7:**
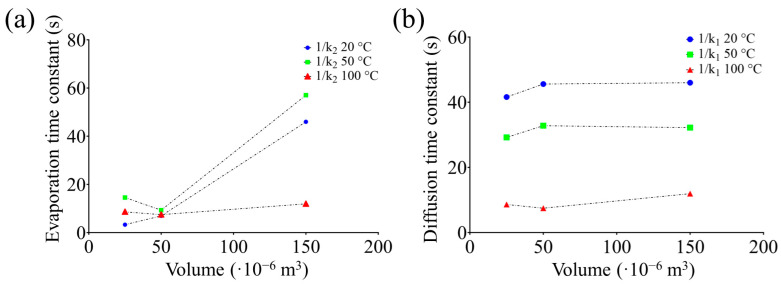
Changes in emissions dynamics related to 1/*k*_1_ evaporation decay constant (**a**) and 1/*k*_2_ diffusion decay constant (**b**) versus analysis chamber volume and temperature.

The time constant 1/*k*_1_ during the evaporation phase is reported in Figure 7a, which highlights that the increase in the volume of the analysis chamber with the same temperature of desorption resulted in an increase in the coefficient. Considering the diffusion phase, the coefficient 1/*k*_2_ (Table 2) under the same conditions showed values that did not significantly increase as the temperature increased. The diffusion rate was slower because, between the end of the evaporation phase and the beginning of diffusion, a plateau phase occurred in which the VOC molecules had already been released from the zeolite layer.

Comparing the different dynamics related to 1/*k*_2_, temperature does not significantly affect the diffusion process. In fact, as shown in Figure 7b, a similar behavior occurred as the temperature changed. The same conclusion can be drawn by comparing the results of Table 2 with the dynamics of the emission profiles in Figure 6. In particular, the dynamics of the emissive profiles show that increasing the temperature and reducing the volume of the analysis chamber resulted in a rapid evaporation phase (fast growth time), while during the dynamics of the diffusion process, a slow recovery time occurred. In addition, as expected, at 100 °C faster evaporation and diffusion processes occurred, suggesting that a higher temperature can be useful for a rapid analysis. Obviously, attention should be paid to the nature of the molecule, especially if it can be altered by the thermal treatment.

**Figure 8:**
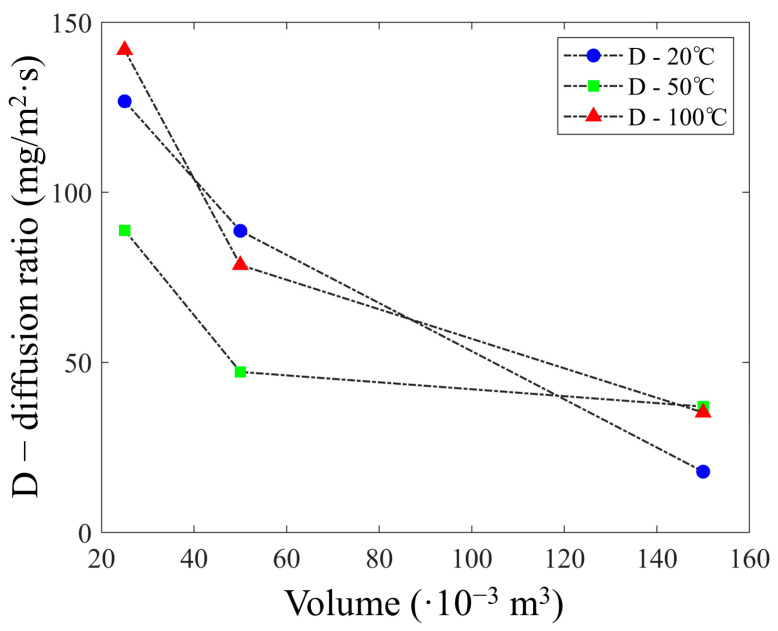
Diffusion ratio trend of propionic acid molecules versus volume of analysis chamber and temperature.

Another factor that has been focused on is the variation in the diffusion ratio (D) in relation to the temperature and the volume of the analysis chamber. As can be seen in Figure 8, by increasing the temperature and decreasing the volume of the analysis chamber, the diffusion ratio can be increased (Table 3) as the area/volume ratio increases. This results in a higher sensitivity of the detection system. Since D is a quantitative index of VOCs, looking at the emission profiles in Figure 6, it is possible to show an increase in the peak concentrations that turns out to be proportional to the temperature and inversely proportional to the chamber volume.

**Figure 9:**
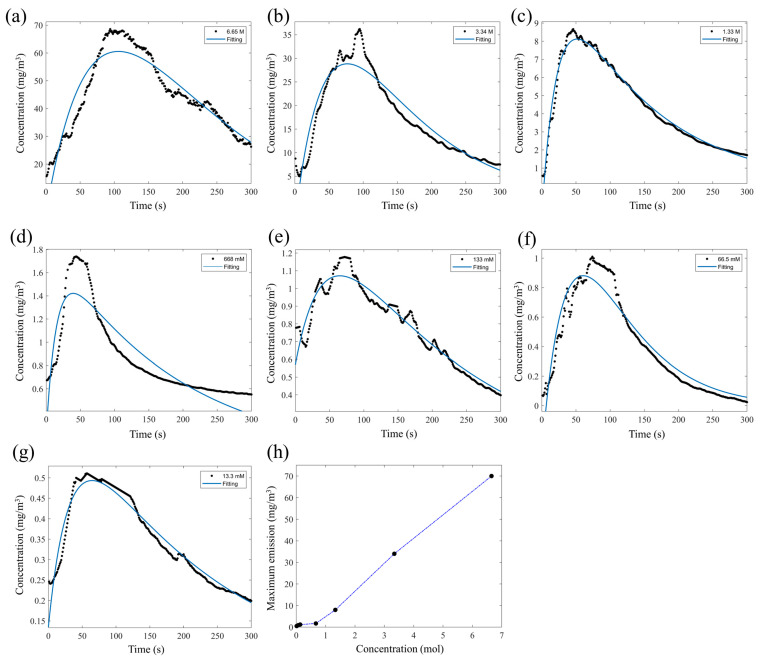
Emission dynamics and regression model using propionic acid at different concentrations in the range of 6.65 M–13.3 mM (**a**–**g**). Linear behavior of zeolite layer between peaks of emission versus change in sample concentration (**h**).

These results highlight that a proper sizing of the analysis chamber allows a higher area/volume ratio and the use of samples with low VOC concentrations without reducing the signal intensity. These factors result in a better analysis phase, obtaining a more accurate signal and faster analysis times. Based on these results, it has been defined that the best configuration to conduct a correct analysis of VOCs using the PID-zeolite system is to place the zeolite layer for an adsorption time of 15 min, followed by a thermal desorption process at a temperature of 100 °C and using a volumetric analysis chamber equal to 25 × 10^−6^ m^3^. Since the zeolite layer is capable of being selective for propionic acid molecules (Figure 3), other tests were performed to define the thermal desorption dynamics of propionic acid using an analysis chamber with a volume of 50 × 10^−6^ m^3^, a temperature of 100 °C, and different sample concentrations. These tests resulted in a similar trend, as shown in Figure 9.

By reducing the concentration of propionic acid in the sample from 6.65 M down to 13.3 mM, the emissivity profiles were characterized by a trend that was linear between the desorption and the change in the sample concentration. Considering the maximum peaks of emission related to the different sample concentrations, as reported in Figure 9a–g, this result demonstrated a linear behavior of the zeolite layer, as shown in Figure 9h. This allowed us to obtain the reproducibility of the emission profile over several adsorption cycles and reuse the zeolite layer. The first results showed that the concentration of volatile organic compounds (VOCs) detected was strongly influenced by the volume of the analysis chamber and adsorption time. Specifically, the experiment showed that when the concentration of the sample solution was halved from a 13.36 M pure solution (Figure 3) to 6.65 M (Figure 9) and the volume of the analysis chamber was reduced from 150 × 10^−6^ m^3^ to 50 × 10^−6^ m^3^ while maintaining the same adsorption time, an increase in the concentration of VOCs was detected. This suggests that the reduced volume of the analysis chamber may lead to a higher concentration of VOCs in the air, which can increase the sensitivity of the detection method.

The results evidenced that the parameters used in the evaporation diffusion model fit well if compared with the physical system, obtaining a good agreement between the VOC concentrations and the emissions dynamics that occur inside the analysis chamber. The proposed technique can be adopted, considering the specific analyte and range of concentration, by adapting the evaporation temperature compatibly with the biological characteristics of the target. Moreover, the volume of analysis plays an important role in the diffusion of evaporated molecules and should be considered in accordance with the characteristics of the photoionizer. It has significant potential to improve the characteristics of commercial or custom PIDs for the detection of VOCs in samples, even at low concentrations. 

## 4. Conclusions

The approach used in this study represents an alternative method for the analysis of VOCs that employs a detection and analysis system based on a zeolite-modified PID system, representing an analytical separation technique based on the physical adsorption and thermal desorption processes of VOCs. A double exponential mathematical model was used, which can describe the evaporation and diffusion processes that occur through a zeolite layer during the thermal desorption process. In general, this model allows us to understand how the evaporation and diffusion processes are influenced by environmental factors, types of materials, the sizing of the analysis system, etc., consequently affecting the analysis of VOCs. 

The use of this analytical technique by combining PID and zeolite made it possible to highlight the ability of the zeolite layer to discriminate between propionic and succinic acid molecules, demonstrating that zeolite 4A is selective only for propionic acid molecules. Meanwhile, considering the parameters provided by the mathematical model, it was possible to define the adsorption dynamics. These were defined by considering the highest value obtained by summing the *E/k*_1_ + *D/k*_2_ ratios, which can quantify the total amount of propionic acid molecules previously adsorbed within the zeolite and subsequently released into the volume during the thermal desorption process. Using a linear regression, the results showed that the time that maximizes the adsorption of propionic acid molecules by the zeolite layer is 15 min. Outside this adsorption interval, a progressive reduction in the VOC concentration was shown, which is important to define and control the adsorption time to ensure reliable and accurate VOC emission.

Another aspect highlighted in this work is how the evaporation process is strongly influenced by temperatures and the volume of the analysis chamber. In fact, considering the same desorption temperature and increasing the volume of the analysis chamber, there is an increase in the decay of the evaporation constant (*k*_1_), resulting in a slow evaporation time (growth time). The optimal temperature allows us to release and detect the true VOC concentration.

Similarly, the equation describing the diffusion process showed that the result is strongly influenced by the volume of the analysis chamber. In fact, increasing the temperature and reducing the volume of the analysis chamber increases the diffusion ratio (D) by increasing the area/volume ratio. In this case, D represents a quantitative indicator of the VOCs present in the analysis chamber, which allows a higher detection resolution of the system. Furthermore, it has been found that the dynamics related to the diffusion decay constant (1/*k*_2_) do not appear to be strongly influenced by temperature.

The results presented in this paper demonstrate that this technique for VOC analysis can provide a high selective capacity, a short analysis time, and a high resolution of the concentrations of molecules present in small samples. It can achieve results comparable to those obtained using the gold-standard analysis techniques (e.g., GC, MS, etc.).

## Figures and Tables

**Table 1:** Parameters extracted by mathematical regression model of MATLAB^TM^ that describe the dynamics during the adsorption and desorption processes of the zeolite layer.

Time (min)	*L* (m^2^/m^3^)	*E* (mg/m^2^·s)	*k*_1_ (s^−1^)	*n* (L/s)	*D* (mg/m^2^·s)	*k*_2_ (s^−1^)	*E/k*_1_ (mg/m^2^)	*D/k*_2_ (mg/m^2^)	R^2^
5	0.21	150.0	0.46	0.004	110.1	0.46	322.9	237.1	0.90
15	0.09	94.2	0.10	0.009	87.5	0.10	979.2	909.5	0.83
30	0.12	134.7	0.15	0.004	129.7	0.15	897.4	864.1	0.98
60	0.05	4.9	0.01	0.008	2.0	0.01	618.3	248.9	0.87

**Table 2:** Emission and diffusion decay constants of propionic acid molecule versus temperature and volume.

Volume (×10^−6^ m^3^)	1/*k*_1_ (20 °C) (s)	1/*k*_1_ (50 °C) (s)	1/*k*_1_ (100 °C) (s)	1/*k*_2_ (20 °C) (s)	1/*k*_2_ (50 °C) (s)	1/*k*_2_ (100 °C) (s)
25	3.30	14.56	8.60	41.60	29.20	8.61
50	6.94	9.31	7.47	75.58	52.81	7.47
150	45.99	57.04	11.93	45.99	32.20	11.93

**Table 3:** Diffusion ratios of propionic acid molecules vs. temperature and volume.

Volume (10^−6^ m^3^)	D (20 °C) (mg/m^2^·s)	D (50 °C) (mg/m^2^·s)	D (100 °C) (mg/m^2^·s)
25	126.80	88.84	141.90
50	88.67	47.17	78.61
150	17.86	36.97	35.22

## Data Availability

The data are contained within the article.
